# Electrochemically actuated microelectrodes for minimally invasive peripheral nerve interfaces

**DOI:** 10.1038/s41563-024-01886-0

**Published:** 2024-04-26

**Authors:** Chaoqun Dong, Alejandro Carnicer-Lombarte, Filippo Bonafè, Botian Huang, Sagnik Middya, Amy Jin, Xudong Tao, Sanggil Han, Manohar Bance, Damiano G. Barone, Beatrice Fraboni, George G. Malliaras

**Affiliations:** 1 Electrical Engineering Division, Department of Engineering, University of Cambridge, Cambridge, UK; 2 Department of Physics and Astronomy, University of Bologna, Bologna, Italy; 3 Department of Clinical Neurosciences, University of Cambridge, Cambridge, UK; 4 Department of Nano-Bioengineering, Incheon National University, Incheon, Republic of Korea

**Keywords:** Electronic devices, Actuators, Polymers, Biomedical engineering, Electrical and electronic engineering

## Abstract

Electrode arrays that interface with peripheral nerves are used in the diagnosis and treatment of neurological disorders; however, they require complex placement surgeries that carry a high risk of nerve injury. Here we leverage recent advances in soft robotic actuators and flexible electronics to develop highly conformable nerve cuffs that combine electrochemically driven conducting-polymer-based soft actuators with low-impedance microelectrodes. Driven with applied voltages as small as a few hundreds of millivolts, these cuffs allow active grasping or wrapping around delicate nerves. We validate this technology using in vivo rat models, showing that the cuffs form and maintain a self-closing and reliable bioelectronic interface with the sciatic nerve of rats without the use of surgical sutures or glues. This seamless integration of soft electrochemical actuators with neurotechnology offers a path towards minimally invasive intraoperative monitoring of nerve activity and high-quality bioelectronic interfaces.

## Abstract (web summary)

Electrode arrays for neurological diagnosis and treatment carry a risk of nerve injury. Nerve cuffs with tiny voltage-controlled shape-reconfigurable electrode arrays have been reported, allowing active wrapping around delicate nerves.

## Main

Peripheral nerve interfaces are increasingly used in bioelectronic medicine interventions to target chronic neuropathic pain, movement disorders, metabolic diseases and closed-loop control of prosthetic limbs^1,2^. Compared with non-invasive strategies, implantable nerve interfaces provide direct access to target nerve fibres, allowing the precise and selective modulation and recording of nerve activities. Nerve cuffs, which circumferentially wrap around a nerve, have attracted considerable interest because of a lower risk of nerve damage compared with nerve-penetrating electrodes^3^. Nevertheless, current cuff electrodes utilize flexible two-dimensional planar structures or thick silicone-rubber-based three-dimensional split-cylinder electrodes with predefined, rigid architectures^4^. The implantation of these devices necessitates complex surgical procedures to ensure proper attachment around the nerve, involving the frequent use of sharp surgical tools and fixation with sutures or adhesives^5^. Together with the mechanical and geometrical mismatch between devices and nerves, they inevitably lead to irreversible nerve damage, particularly for small nerves or at complex anatomical locations^6^. Moreover, existing tubular implants lack adaptability to nerves with different diameters and are typically short (length of a few millimetres), offering limited interfacing along the length of a nerve^7^. Once anchored, they cannot be repositioned to explore optimal electrical contact sites or to address progressively weakening signals in chronic interfaces. In addition, nerve surgeries in clinic require better intraoperative nerve-monitoring electrodes that offer continuous neurophysiological recording throughout the surgery process to preserve the structure and function of nerves^8,9^. Recent work on flexible and even stretchable thin-film nerve cuffs addresses some of these issues^3,8,10^; however, innovations in minimally invasive implantation and high-quality nerve recording and stimulation at optimal electrical sites are urgently needed.

A promising strategy to alleviate this challenge relies on the exploration of soft robotic actuators. Recent advancements in soft materials and processing techniques have greatly accelerated the development of soft robotics that can safely interface with different tissues, finding applications in wearables, healthcare, surgical tools and human–machine interfaces^11–16^. Examples include soft robotic vascular microcatheters for minimally invasive surgeries that can be omnidirectionally steered and navigated^17–19^. These developments have also led to self-shaping peripheral nerve cuffs, relying on hydrogel swelling on wetting^20^ or shape memory materials responding to body temperature^21–23^. These nerve interfaces, however, lack the ability to reverse and thus reprogram their configuration in the body environment, are suitable only for single use and face limitations on the geometries and functions that can be achieved^24^. Thus far, the development of nerve cuffs that allow the continuous and reversible control of complex three-dimensional shapes remains elusive.

We hypothesized that conducting polymers could effectively address these limitations, primarily due to their controllable volumetric expansion or contraction in response to safe, low-voltage stimuli^25–27^. This behaviour is achieved through a reversible electrochemical process occurring in ion sources, such as aqueous electrolytes, which makes conducting polymers particularly attractive for applications in biology and medicine. Unlike many other materials or systems that require extensive protection from liquids in case of failure, conducting polymers offer inherent safety and design flexibility in a saline environment^28,29^. A notable example is the construction of bilayer structures, where a conducting polymer is combined with a passive material to induce bending movements in response to electrical signals^30–32^. Such assemblies have been demonstrated for accomplishing tasks such as grasping small objects, acting as sealable microvial lids^33^ or controlling catheters^34^. However, their integration into advanced bioelectronic implants remains unexplored, and practical applications in a complex in vivo environment have not been validated yet. Here we introduce soft robotic, thin-film bioelectronic peripheral nerve cuffs that integrate tens of distributed high-resolution microelectrodes and a conducting-polymer-based bilayer actuator that can be controlled by programmable electrical inputs.

## Actuator design and fabrication

**Fig. 1:**
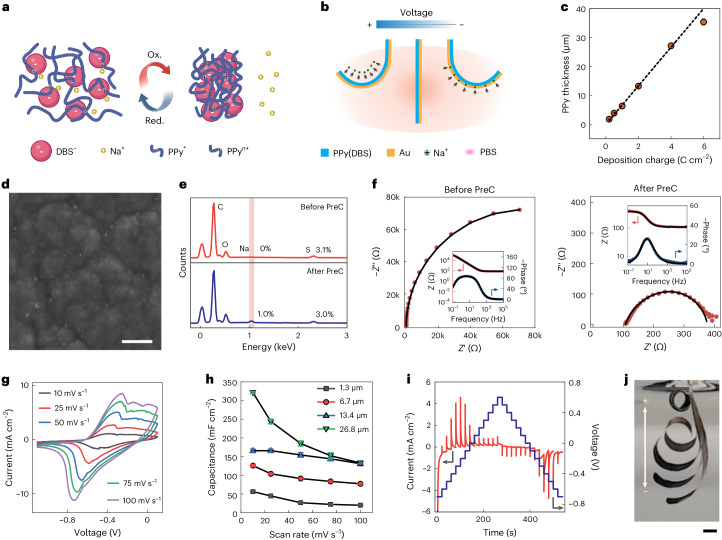
Working principle, fabrication and characterization of electrochemical actuators. **a**, Schematic showing a large volume change in PPy(DBS) in an electrochemical process, induced by the transportation of small cations such as Na^+^. **b**, Reversible bending motion of a PPy(DBS)/Au bilayer actuator back and forth perpendicular to the film surface in a PBS solution. **c**, PPy films with varied thicknesses are fabricated using the galvanostatical method, and the thickness shows a linear relationship with deposition time. Data are shown as mean ± s.d. (*n* = 3). **d**, Scanning electron microscopy images of PPy after electrochemical precondition in NaDBS. **e**, Energy-dispersive X-ray spectroscopy spectra to compare the PPy(DBS) films before and after the preconditioning (PreC). **f**, Nyquist and Bode magnitude and phase-angle plots of PPy(DBS) films before and after the preconditioning. The black lines represent fits to the data using a transmission-line model. **g**, Typical CV curves of 6.7-µm-thick PPy(DBS) collected under various scan rates in PBS. **h**, Calculated areal capacitance from the CV results in **g**. **i**, Multistep amperometry characterization by applying stepwise voltages between −0.7 and 0.6 V in PBS. **j**, Merged sequence of photographs showing large triggered bending motion of the free extremity of a bilayer strip. Scale bars, 500 nm (**d**); 2 mm (**j**). Schematic in **a** is created with BioRender.com. Source data

We start with the design and fabrication of the key actuating component. We select polypyrrole doped with dodecylbenzene sulfonate (PPy(DBS)) as the actuating material due to its substantial volumetric change on electrochemical stimuli. As schematically shown in Fig. 1a, the polymer experiences volumetric expansion when a slightly negative voltage is applied, as solvated cations such as Na^+^ are pulled into the polymer matrix. Conversely, a positive voltage induces the expulsion of cations back to the electrolyte and thus leads to contraction of the polymer. By leveraging this reversible electrochemical process, bilayer configurations formed by PPy(DBS)-coated gold (Au) exhibit controllable bending behaviour (Fig. 1b). We electrochemically deposited PPy(DBS) on Au-coated parylene C (PaC) films by applying a constant current of 2 mA cm^–2^ in a 0.1 M NaDBS/0.1 M pyrrole solution (Supplementary Fig. 1). During the film deposition, negatively charged bulky anions DBS^−^ are incorporated into the polymer matrix to maintain the overall charge neutrality. Overall, the film thickness increases linearly with the amount of deposition charge^35,36^, with an increment of approximately 6.7 µm per C cm^–2^ (Fig. 1c). As the film thickens, it tends to become increasingly non-uniform, undergo compaction and experience a density increase. This could lead to a minor deviation, manifesting as a lower thickness than that predicted by the linear fit^37^.

We precondition the films by performing cyclic voltammetry (CV) scans at 10 mV s^–1^ in 0.1 M NaDBS to fully activate PPy(DBS) (Supplementary Fig. 2 and Supplementary Section 1). Initially, the device was flat, but after a few CV scans, it transitioned into a curled state, accompanied by an irreversible current, particularly noticeable in the first cycle. This behaviour might be attributed to the entrapment of some cations in the polymer matrix, causing structural changes in the polymer. The preconditioning ultimately resulted in a fully curled state even in the absence of any applied voltage. Scanning electron microscopy imaging (Fig. 1d and Supplementary Fig. 3) reveals the presence of nanoparticles in the valleys of the uneven substrate after the treatment, in contrast to the clean surface before the preconditioning. Energy-dispersive X-ray spectroscopy spectra (Fig. 1e) demonstrate the absence of sodium before preconditioning, whereas an apparent sodium peak, with an intensity equal to one-third that of sulphur, is observed after the treatment. Hence, we infer that the nanoparticles shown in Fig. 1d correspond to sodium trapped in the polymer, which is also consistent with the notable reduction peaks (Supplementary Fig. 2). Our analysis of the electrochemical impedance of PPy(DBS) films using a transmission-line model indicates that increased charge transfer to the material occurs after preconditioning (Fig. 1f, Supplementary Section 2, Supplementary Fig. 4 and Supplementary Table 1). We then investigated the electrochemical properties of the PPy(DBS) film in phosphate-buffered saline (PBS) (Fig. 1g–i and Supplementary Fig. 5). Supplementary Section 3 provides a detailed discussion. In Fig. 1j, a merged sequence of photographs demonstrates the process of a thin-film strip curling into spirals, highlighting the large strain enabled by a substantial volume change in PPy(DBS) and the low stiffness of the entire configuration. Supplementary Video 1 showcases this demonstration. This demonstration was conducted in PBS, whereas there is no current flow and actuation observed in deionized water (Supplementary Fig. 6).

## Actuator evaluation

**Fig. 2:**
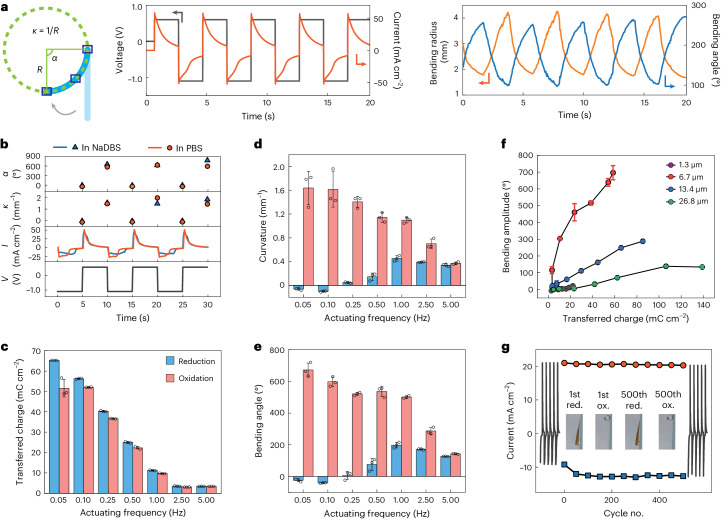
Electro–chemo–mechanical evaluation. **a**, Schematic of the side view of a device’s bending behaviour (in blue colour) and a kinematic assessment model (in green colour) constructed on the basis of the constant-curvature approximation (left). The two extremities of the film together with a random point in between are selected to fit the bending angle (*α*), bending radius (*R*) and curvature (*κ*). Computer vision to track the time-variant deformation of an exampled device in response to the stimulation of square-wave voltages (middle and right). The thickness of PPy(DBS) is 13.4 µm. **b**, Performance comparison of devices tested in NaDBS and PBS. The thickness of PPy(DBS) is 6.7 µm, and square-wave voltages between −1.1 and 0.6 V are applied. The devices can curl into spirals, where each circle corresponds to a bending angle of 360°. **c**–**e**, Transferred charge amount (**c**), curvature (**d**) and bending angle (**e**) as a function of the actuating frequency in PBS. The thickness of PPy(DBS) is 6.7 µm. Data are shown as mean ± s.d. (*n* = 3). **f**, Evaluation of bending amplitude as a function of transferred charge. The same voltages were applied to all the samples. Data are shown as mean ± s.d. (*n* = 3). **g**, Robustness evaluation during 500 repeated actuating cycles. The red and blue curves represent the peak positive and negative currents recorded throughout the 500 actuating cycles, with data collected at intervals of every 50 cycles. Source data

Next, we investigated the actuating performance of PPy(DBS)/Au/PaC actuators with a simple rectangular shape. We use a kinematic model to assess the bending movement on the basis of the constant-curvature approximation (Fig. 2a). Specifically, the two extremities of the strip together with a random point in between are utilized to fit the bending, generating evaluation parameters including bending angle (*α*), bending radius (*R*) and curvature (*κ*). Square-wave voltages alternating between −1.1 and 0.6 V were applied, and the device’s movement was recorded using a camera and analysed via computer vision. The devices demonstrate an immediate response to the stimulation, and the movements exhibit excellent reversibility and repeatability (Fig. 2a and Supplementary Video 2). We then tested the actuators in PBS to reveal their performance in physiologically relevant settings. The behaviour in NaDBS—a standard electrolyte to characterize conducting polymer actuators—was analysed, too, for reference purpose. The devices exhibit remarkable bending performance and are even capable of curling into spirals in both solutions. We quantified the kinematic features, considering each spiral as a bending angle of 360°. Bending angle, radius and curvature are designated as positive when the device bends towards the PPy(DBS) side, and negative otherwise. As shown in Fig. 2b, the films become flattened, exhibiting a nearly zero bending angle and curvature after being subjected to a negative voltage. Subsequently, on the application of a positive voltage, the films rapidly transform into spirals. They exhibit an average curvature of 1.6 mm^−1^ and average bending angles of up to 682° and 597° in NaDBS and PBS, respectively. We further explored the response time of the devices by applying square-wave voltages with multiple frequencies in the range between 0.05 and 5.00 Hz. The amount of transferred charge, bending curvature and angle for both reduction and oxidation processes were calculated (Fig. 2c–e). Overall, higher frequencies involve fewer cations participating in the reaction and therefore lead to a gradual reduction in the bending amplitude. This phenomenon is attributed to the time required for cations to move through the polymer matrix. The performance of devices with varying PPy(DBS) thicknesses exhibits a consistent trend (Supplementary Fig. 7). Generally, the bending amplitude increases with the transferred charge amount, as well as being highly dependent on the thickness of PPy(DBS) (Fig. 2f). As the PPy(DBS) layer is relatively thin compared with the substrate, the bending amplitude increases with an increase in PPy(DBS) thickness. This tendency is attributed to the higher efficiency of thicker films regarding charge-induced swelling, which may arise from osmotic swelling, conformational changes and Coulombic repulsion of the polymer chains^37^. As the thickness continues to increase, the stiffness of the bilayers undergoes a notable increase, leading to a restriction in the bending movement. The interplay between the enhanced bending force and increased stiffness reveals an optimal thickness of 6.7 µm, which demonstrates the maximum bending amplitude when coupled with 2 µm PaC substrates. Moreover, we confirm the robust stability and reliability of the actuators through a cyclic test involving 500 repeated stimulation cycles (Fig. 2g).

## Design of nerve cuff electrodes

**Fig. 3:**
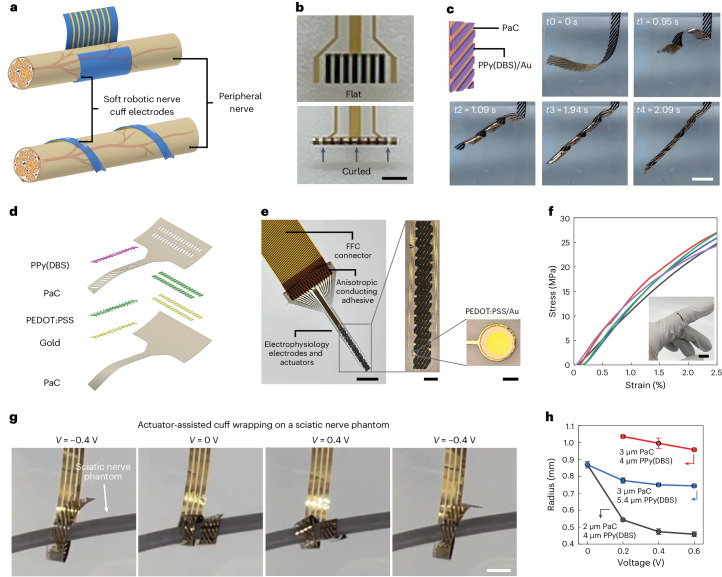
Soft-actuator-integrated nerve cuff electrodes. **a**, Examples of the proposed nerve cuffs with targeted shape transformation paths and bending curvatures that are adaptive to nerve dimensions and surgery needs, realized through the prescribed arrangement of actuator elements. **b**, Optical micrographs of a flat cuff device that can be bent into curls with a bending radius of 170 µm. The cuff integrates two recording electrodes at the left and right edges, with aligned micro-striped actuators in between. **c**, Photograph series showing the swift actuation following a helical path in 2.1 s, enabled via the design of asymmetrically patterned actuating elements. The black-coloured strips are PPy(DBS)/Au. The applied voltage is 0.6 V. **d**, Exploded device render showing each layer of the robotic thin-film nerve cuffs. Here, for simplicity, the depiction excludes the conducting Au tracks within the Au layer. **e**, Photograph of the device bonded to a flat flexible cable (FFC) connector showing the overall structure. The optical micrographs on the right show a detailed design of the implanted interfacing section. **f**, Stress–strain curves for thin-film devices composed of 1.95 μm PaC, 10 nm Ti, 100 nm Au and 6.70 μm PPy(DBS). Five samples were tested. The photograph in the inset shows a microfabricated nerve cuff conforming to a finger, demonstrating the high conformability of the device. **g**, Photograph sequence showing a nerve cuff wrapping on a 1.4 mm nerve phantom in PBS. A simplified cuff with three microelectrodes was used for this demonstration. **h**, Comparison of the radius of the helical structures under different voltages. Devices with varied thicknesses of PaC and PPy were measured. The bending radius measured for the 3 μm PaC and 4 μm PPy(DBS) sample at 0 V exceeds 5 mm, which surpasses our plotting scope and thus is omitted from this plot. Data are shown as mean ± s.d. (*n* = 3). Scale bars, 1 mm (**b**); 5 mm (**c**); 5 mm, 1 mm, 50 µm (**e** (from left to right)); 1 cm (**f**); 2 mm (**g**). Source data

Our soft robotic peripheral nerve cuffs consist of micropatterned actuating elements for on-demand shape morphing, surrounded by distributed electrophysiology electrodes. In Fig. 3a, we show two illustrations: one depicting the gentle holding of a nerve, whereas the other demonstrates a helical wrapping around the nerve. The latter approach enables adaptation to nerves with varying diameters, avoiding communication issues that often occur in conventional cuffs due to poor electrode–nerve bundle alignment^38^. The actuators are essentially electronic elements that share the same lithography fabrication processes as the microelectrode arrays. This seamless integration leaves room for us to engineer the shape and distribution of the actuators for custom shape-changing needs. For example, the vertical alignment of the actuator elements minimizes bending towards the perpendicular direction, allowing large bending only along the alignment direction of electrodes with an achievable bending radius as small as 170 µm (Fig. 3b). It is noteworthy that we achieved helically wrapped nerve cuffs by engineering the asymmetric distribution of the actuator elements. As shown in Fig. 3c and Supplementary Video 3, the integration of tilted Au/PPy(DBS) elements enables rapid transformation from the original shape to a helical configuration, forming four turns in 2.1 s.

We then constructed helically actuatable nerve cuffs using this asymmetric structure. An exploded view of the cuff is schematically depicted in Fig. 3d, and Methods provides a detailed description of the fabrication process. The overall structure of the devices is shown in Fig. 3e, with magnified optical micrographs providing details of the actuators and microelectrode arrays. The custom interfacing part, which includes the actuators and microelectrodes, measures 2.1 mm in width and 10.7 mm in length. In particular, all the functional elements share a single set of flexible electrical contacts and connecting lines, eliminating the need for additional complex and rigid controlling components typically found in conventional actuation mechanisms. This simplification reduces the risks of mechanical mismatches and facilitates the miniaturization process. In static tensile tests (Fig. 3f), our thin, laminated structure exhibited a modulus of 1.24 GPa, closely aligned with that of PaC (1.13 GPa), indicating minimal alteration by the thin metal layers. Despite PaC-based devices being stiffer than nerve tissue, their ultrathin form factor imparted remarkable flexibility^39^. We estimated their bending stiffness using a model derived from the Föppl–von Kármán plate theory^40^. The resulting bending stiffness is 21 Pa mm^3^, equivalent to a single PPy(DBS) layer with a thickness of 11.4 μm or a single poly(dimethylsiloxane) layer with a thickness of 40.0 μm. Supplementary Section 4 provides the detailed calculations. This low stiffness effectively addresses the implant–tissue mechanical mismatch, promoting a more intimate and stable interface and substantially reducing foreign body reaction driven by tissue damage. We validated the cuffs’ capability of self-wrapping around nerves in vitro using a simplified setup comprising an insulated wire as a sciatic nerve phantom submerged in a PBS solution and placed over an agarose gel. A small gap was maintained between the wire and the gel, allowing the device to pass through. As demonstrated in Fig. 3g and Supplementary Video 4, on the stepwise application of voltages from −0.4 to 0.4 V, the device slowly wrapped around the phantom and gradually tightened until the formation of a firm grasp, and released on the application of −0.4 V. The wrapping radius of the device was controlled by the thicknesses of the PaC and PPy(DBS) layers (Fig. 3h, Supplementary Section 5 and Supplementary Fig. 8).

## In vivo validation of the nerve cuffs

**Fig. 4:**
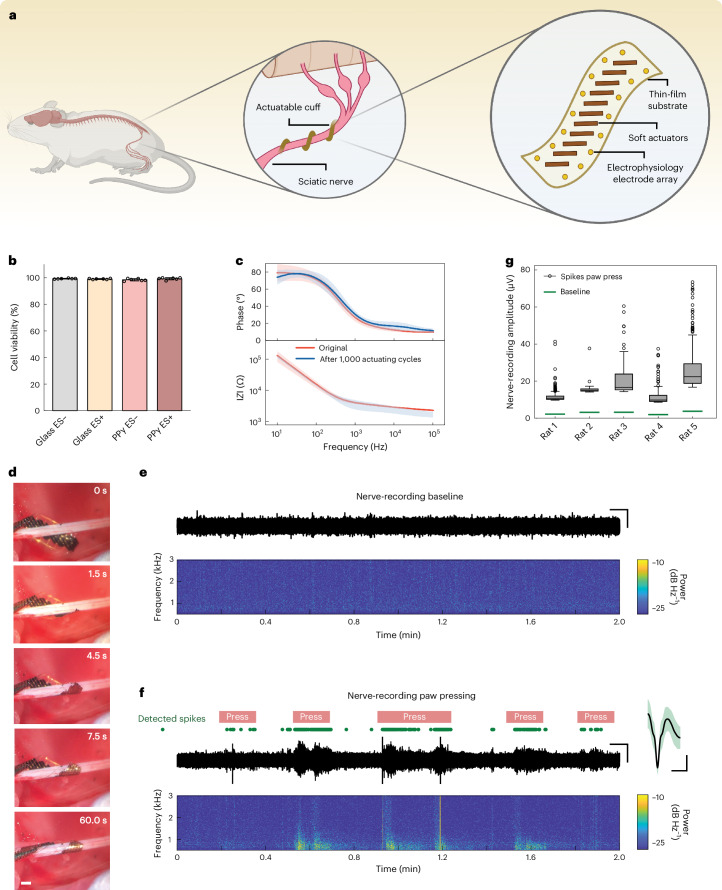
Implantation and in vivo validation of the soft-actuator-assisted peripheral nerve cuffs for minimal invasive surgeries. **a**, Conceptual schematic of a flexible nerve cuff actively wrapping around a sciatic nerve for an intimate interface without sutures. **b**, Cell viability analysis of PPy(DBS) and control groups of glass slides. ES– and ES+ mean without and with the application of voltage stimulation, respectively. Data are shown as mean ± s.d. (*n* = 6). **c**, Electrochemical impedance magnitude (bottom) and phase (top) of the neural-recording microelectrodes ahead of and after 1,000 actuating cycles. The solid lines represent mean values and shaded area represent the s.d. **d**, Self-wrapping of the nerve cuff around a sciatic nerve on the switching of voltage from −0.5 to 0 V. **e**, Recording baseline noise from an electrode of the soft-actuator-assisted cuff implanted into the sciatic nerve of an anaesthetized rat, shown as a trace (top) and spectrogram (bottom). **f**, Trace and spectrogram of a representative recording from the sciatic nerve during sensory stimulation in the form of pressing of the hindpaw. Approximate periods of paw presses are indicated as red lines. The green circles indicate the recorded spikes (amplitude 4.5 times above the background noise). The average spike waveform is shown on the right. **g**, Quantification of noise (green line) and recorded spike (box-plot) amplitudes in response to paw presses, for five implanted actuator-assisted cuffs on five rats: *n* = 924 (rat 1), 34 (rat 2), 63 (rat 3), 109 (rat 4), 411 (rat 5) spikes. Box plots display the data median (centre line), upper and lower quartiles (bounds of box), 1.5 times the interquartile range (whiskers) and outlier values beyond this range (circles). Scale bars, 1 mm (**d**); 5 s, 20 µV (**e**); 5 s, 20 µV, 1 ms, 5 µV (**f** (from left to right)). Schematic in **a** created with BioRender.com. Source data

We, thus, chose the combination of 2-µm-thick PaC and 4-µm-thick PPy(DBS) as the final design in the following in vivo validation on rat sciatic nerves, where 28 poly(3,4-ethylenedioxythiophene) polystyrene sulfonate (PEDOT:PSS)/Au microelectrodes are integrated for nerve activity recordings (Fig. 4a). To assess the cell viability of PPy(DBS) before in vivo validation, we performed a live/dead cell assay using SH-SY5Y cells seeded on PPy(DBS) and glass slides as a control. We applied 300 repeated cycles of ±0.5 V stimulation to study the effect of electrochemical actuation, and the cell viability was evaluated after 72 h. All the PPy(DBS) samples exhibit remarkable cell viability, with values around 99%, similar to the control groups on glass slides (Fig. 4b and Supplementary Fig. 9). In addition, the incorporation of PEDOT:PSS in the electrophysiology microelectrodes results in a low impedance (4.1 ± 0.4 kΩ at 1 kHz in PBS) and negligible increases even after 1,000 large bending cycles (Fig. 4c).

On the completion of configuration design and characterization, we placed the cuff electrodes on the sciatic nerve of anaesthetized rats for in vivo validation. A PEDOT:PSS-coated stainless steel wire was used as the reference electrode, and voltages were applied between this electrode and the actuators to initiate the shape transformation. The devices exhibit a default curling shape before implantation. The device was initially opened by applying a voltage of −0.5 V, which allowed us to manually position it adjacent to the nerve. Subsequently, on the removal of the applied voltage, the device gradually self-wrapped around the nerve in a helical manner. This reversible actuation process allowed for repeated retrieval and adjustment until achieving the desired three-dimensional conformal interface (Fig. 4d). We performed four to five adjustments using this method. We then assessed the quality of the interface achieved without any fixation glue or sutures of the device by recording nerve activity evoked by pressing of the paw. Representative traces of the recordings at rest and under paw pressing are displayed in Fig. 4e,f, respectively. The recorded signal was band-pass filtered (0.6–3.0 kHz) and notch filtered to reduce mains noise. The implanted devices were able to record bursts of spikes associated with paw-press sensory stimuli, yielding low baseline noise and high, stable spike amplitude in their recordings (Fig. 4f,g). During the test in Fig. 4f, the paw was manually held by the surgeon. Minor spikes were observed before paw presses due to slight movements of the surgeon’s hand. Once the recording was completed, we applied −0.5 V to the actuators to gently loosen the interface, allowing for an easy extraction of the device. Attempting to directly remove this closed structure without loosening it first requires the application of a larger force to overcome friction and capillary forces between the device and the nerve. This force, in turn, can markedly compress the tissue, leading to nerve damage, whereas the application of a slightly negative voltage to loosen the structure represents a biocompatible technology that eliminates such injuries. These results highlight the potential of soft-actuator-integrated nerve cuffs in facilitating more flexible and precise implantation and explantation, as well as achieving a conformal interface with delicate nerves.

The integration of soft electrochemical actuators into thin-film bioelectronics enabled a new type of flexible—and importantly—low-voltage shape-actuated nerve cuff electrodes for minimally invasive nerve-interfacing surgeries. Leveraging conventional microfabrication techniques, we created thin-film devices that incorporate precisely patterned actuator elements and microelectrode arrays in a single structure. This electrically driven soft robotic electrode eliminates the need for additional bulky and rigid actuators and the accompanying complicated control systems commonly used in other approaches. The shape transformation is realized by the virtue of the reversible transport of solvated cations in response to the applied voltages. To ensure their safe use in biomedical situations, we have substantially reduced the voltage required for actuation to very low values. Although this may result in lower actuating forces, the micrometre-thick film structures allow desirable large actuating strains. We demonstrated different shape-transforming modes by designing the distribution of the actuating elements. For instance, devices with aligned actuators along the length can easily bend into spirals without extra strain from the perpendicular direction. Additionally, complex helical shapes can be achieved by breaking structural symmetries. Our study highlights that the thickness of each layer must be carefully engineered to optimize the performance for specific application settings. Moreover, we envision finite-element analysis as a valuable tool for further investigations into the interplay among various design factors governing helical actuation behaviour. Understanding the relationship between cation diffusion and bending amplitude, particularly under conditions involving extreme bending, is a key aspect of this exploration. However, challenges persist in establishing a reliable model to describe the mechanical behaviour of PPy(DBS), including the need for the precise understanding and description of the actuation strain gradient and elastic modulus along the PPy(DBS) thickness direction^41^. Acute animal trials demonstrate that the actuators can facilitate self-wrapping of the device around the nerve, establishing an intimate interface for reliable signal recording. This approach eliminates the need for additional fixation operations that are commonly used in traditional nerve cuffs. Unlike other reported smart nerve cuffs that rely on one-time-use shape-changing mechanisms, our devices can withstand hundreds of cycles of repeated folding and unfolding within the body environment. This resilience allows surgeons to fine-tune the interface for optimal signal quality or to locate specific spiking units. Maintaining a minimal clamping force for nerve cuffs is crucial to avoid injuries to delicate nerves. Through precise engineering, our cuff electrodes bend with an appropriate radius, striking a balance between intimate contact for effective recording and preventing compression of nerves. During cuff placement, we can slowly reduce the bending radius by gradually increasing the applied voltage. Additionally, we have the flexibility to pause the wrapping at any point to adjust the nerve cuff and observe the interface. Besides, it enables easy device extraction without causing damage to the nerves. In our current design, all the actuating elements are simultaneously controlled by the same voltage, whereas we envision future developments where discrete actuator elements can be engineered for the independent control of each section, enabling more complex shape transformations. These soft actuators can find immediate application in intraoperative nerve monitoring, for example, the monitoring of nearby nerves during tumour extraction, where a short-term, reconfigurable and removable interface is needed. In other clinical scenarios, such as vagus nerve stimulation, the ability of these implants to be repositioned during implantation for optimal contact would be a valuable feature.

## Methods

### Microfabrication of nerve cuffs

The nerve cuffs were fabricated using standard photolithography techniques with the following steps: initially, a micrometre-thick layer of PaC was deposited on silicon wafers through chemical vapour deposition. Subsequently, the PaC layer was coated with AZ 5214E photoresist (Merck) by spin coating at a speed of 4,000 r.p.m. for 30 s. After a pre-baking step at 110 °C for 2 min and cooling down, the wafers were exposed to ultraviolet light (7 s, 80 mJ cm^–2^) using a mask aligner (Karl Suss Contact Mask Aligner MA/BA6). Afterwards, a second baking step at 110 °C for 2 min was carried out, followed by flood exposure to ultraviolet light (20 s, 80 mJ cm^–2^). The substrates were developed in AZ 351B developer/deionized (DI) water (1:4) for 15 s. Subsequently, they were rinsed with DI water and air dried. All the substrates were inspected with a ZEISS Axioscope (A1) to ensure complete development. Next, the substrates were activated with O_2_ plasma and baked at 80 °C for 5 min before the deposition of 10 nm titanium (Ti) layer and 100 nm gold (Au) layer using a Lesker electron-beam evaporator. Liftoff was performed by soaking the substrates in acetone for 10 min, followed by washing with acetone spray and isopropyl alcohol. A second layer of PaC was deposited to insulate the gold tracks. Photolithography was adopted to pattern the outlines of the individual devices. The substrates were spin coated with AZ 10XT photoresist at 3,000 r.p.m. for 30 s, and baked at 110 °C for 2 min. Ultraviolet exposure for 30 s was carried out, followed by development in AZ 726 developer for 12 min, rinsing with water and air drying. The substrates were etched using reactive ion etching (PlasmaPro RIE 80, Oxford Instruments; 50 s.c.c.m. O_2_, 6 s.c.c.m. CF_4_, 4 s.c.c.m. SF_6_, 180 W plasma power). After rinsing with acetone and isopropyl alcohol and air drying, a 3% Micro-90 detergent solution (Cole-Parmer) was spin coated to form an anti-adhesion layer, followed by the deposition of a third layer of PaC as the sacrificial layer. Subsequently, photolithography was repeated to expose the Au electrodes and contacts using AZ 10XT photoresist, before the final etching of the top two layers of PaC.

### PEDOT:PSS for the electrophysiology microelectrodes

A modified PEDOT:PSS solution consisting of PEDOT:PSS (Clevios PH1000, Heraeus), 5% (v/v) ethylene glycol and 0.05 wt% dodecylbenzene sulfonic acid (Sigma-Aldrich) was prepared and sonicated. Then, 1% (v/v) 3-glycidyloxypropyl trimethoxysilane (Sigma-Aldrich) was added and filtered through a poly(tetrafluoroethylene) filter (0.45 µm pore size) before use. The PEDOT:PSS was spin coated to an ~250 nm film thickness and baked at 120 °C for 1 min. The top sacrificial PaC layer was then peeled off and the substrates were baked further at 120 °C for 1 h to fully crosslink the PEDOT:PSS before soaking in DI water overnight.

### Electrical contacts

The devices were carefully released from the wafer with DI water, transferred onto glass slides and dried on a 55 °C hotplate. Finally, the devices were bonded to a flexible flat cable (Mouser Electronics) using a Finetech bonder (FINEPLACER pico 2, Finetech) and an anisotropic conductive adhesive film (5 µm particulate) (3T Frontiers). During electrical tests, the devices were connected to a custom miniature printed circuit board with preassembled zero-insertion-force connector, a slim-stack connector (for connection to Intan) and wires (for electropolymerization and controlling of actuators).

### Polymerization of PPy(DBS)

PPy(DBS) was electrochemically deposited on Au electrodes using a constant current density of 2 mA cm^–2^ in a three-electrode electrochemical cell connecting to a PalmSens potentiostat. A Pt wire and an Ag/AgCl electrode were used as the counter and reference electrodes, respectively. Subsequently, the films were preconditioned using the CV method. Five cycles of scan between −0.8 and 0.1 V at 10 mV s^–1^ were carried out to precondition the as-fabricated polymer.

### Electrochemical impedance spectroscopy

All the impedance measurements of the recording microelectrodes were taken in a PBS solution, and a platinum electrode was used as the counter electrode. Impedances were scanned between 0.1 Hz and 100 kHz with an input of a 10 mV amplitude sinusoidal voltage.

### Soft actuation and mechanical characterization

To characterize the electrochemomechanical performance, the devices were actuated in an electrochemical cell containing either 0.1 M NaDBS solution or PBS solution using a potentiostat (PalmSens) and a webcam (Logitech) to record the movement of actuators. The bilayer actuators were subjected to stimulation protocols applied through CV and multistep amperometry methods. For small bending movements confined within a full spiral, the movement was analysed using a Python 3.9.12 program coupled with OpenCV 4.5.1 to accurately track the deflection and perform curve fitting. However, when handling larger bending movements surpassing one spiral, the tracking system could not identify the tip’s movement and therefore these data were manually analysed using ImageJ (version 1.46j). The tensile tests were carried out by applying controlled tensile force ramps to devices using a dynamic mechanical analysis instrument (DMA Q800, TA Instruments) in the tensile mode at a ramp speed of 2.5 N min^−1^.

### Live/dead cell staining

SH-SY5Y cells were seeded on polypyrrole substrates at a density of 20,000 cells cm^–2^. Live/dead cell staining using calcein AM and ethethidium homodimer-1 was performed after 72 h to assess viability. Two hours before staining, a voltage of 0.5 V for 1 s followed by −0.5 V for 1 s was applied for a total of 300 cycles (10 min). The images were acquired with a fluorescence microscope, and Fiji 2.10.0 was used to automatically count the cells in images.

### Surgical implantation

All the animal procedures were carried out in accordance with the UK Animals (Scientific Procedures) Act, 1986. Work was approved by the Animal Welfare and Ethical Review Body of the University of Cambridge, and was approved by the UK Home Office (project licence no. PFF2068BC). The experiments were performed under terminal anaesthesia and were conducted on female rats (weight, ~250 g). Surgical implantation of devices was carried out under isoflurane anaesthesia (2.25% v/v in medical oxygen, lowered to 1.5% during electrophysiology recordings). Body temperature was monitored and maintained using a thermal blanket. An incision was made in the anterior upper portion of the leg between the hip and knee joints and the sciatic nerve was accessed. The nerve cuff was in a flat state by applying a low voltage of −0.5 V referred to a grounded PEDOT:PSS-coated stainless steel wire so that the cuff can be manually placed underneath the nerve. Then, the cuff automatically wrapped around the nerve by switching the voltage back to 0 V.

### Nerve electrophysiology recording and analysis

Electrophysiology recordings were carried out by connecting the implant through a custom printed circuit board to a 32-channel stim/recording headstage (Intan Technologies). Data were collected at a 30 kHz sampling rate from the implanted electrodes using an RHS stim/recording system (Intan Technologies), with a ground wire being placed subcutaneously in the contralateral side of the animal. Recordings were performed as the rat hindpaw was gently pressed using a surgical clamp. Paw pressing was repeated at least five times per rat per recording. Electrophysiology analysis and data plotting were performed in MATLAB R2023b and Python. The recorded traces were imported and notch filtered to remove mains noise. They were then band-pass filtered using a fourth-order Butterworth filter with cutoff frequencies of 0.6 and 3.0 kHz. The baseline amplitude of noise was calculated as the standard deviation (s.d.) of the recorded trace. Spikes were identified as negative peaks with amplitude between 4.5 and 20.0 times the noise amplitude.

## Online content

Any methods, additional references, Nature Portfolio reporting summaries, source data, extended data, supplementary information, acknowledgements, peer review information; details of author contributions and competing interests; and statements of data and code availability are available at 10.1038/s41563-024-01886-0.

### Supplementary information

Supplementary Information Supplementary Sections 1–5, Figs. 1–9 and Table 1.

Supplementary Video 1 A thin-film strip curling into spirals.

Supplementary Video 2 Movement tracking of actuators.

Supplementary Video 3 Helical deformation enabled by tilted actuator elements.

Supplementary Video 4 A nerve cuff actively wrapping around a nerve phantom.

### Source data

Source Data Fig. 1

Source Data Fig. 2

Source Data Fig. 3

Source Data Fig. 4

## Data Availability

All the data supporting the findings of this study are available within the Article and its Supplementary Information, as well as from the corresponding author upon reasonable request. Source data are provided with this paper.
