# Supplementary material for: Electrochemically actuated microelectrodes for minimally invasive peripheral nerve interfaces
Source: Nat Mater. 2024 Apr 26;23(7):969–76. doi: 10.1038/s41563-024-01886-0 (PMC11230894; doi:10.1038/s41563-024-01886-0)
Supplement: Supplementary file 1 — Supplementary Sections 1–5, Figs. 1–9 and Table 1. [file 41563_2024_1886_MOESM1_ESM.pdf]

nature materials

Article

https://doi.org/10.1038/s41563-024-01886-0

Electrochemically actuated microelectrodes for minimally invasive peripheral nerve interfaces

In the format provided by the authors and unedited

This PDF file includes:

Supplementary section 1 to 5

Supplementary Figures 1 to 9

Other Supplementary Materials for this manuscript include:

Supplementary Videos 1 to 4

Supplementary section 1. Electrochemical deposition and preconditioning of the PPy(DBS) film

We applied a constant current density of 2 mA/cm^2 in a three-electrode system using a Pt wire and Ag/AgCl as the counter and reference electrodes, respectively. A mixed aqueous solution containing 0.1 M pyrrole and 0.1 M NaDBS was used as the electrolyte. The monomers are polymerized meanwhile DBS^- are incorporated to dope PPy under such a positive current. DBS^- was specifically selected because the molecules are large and remain immobile in the film after fabrication, leaving the movement of small cations in the following electrochemical processes. The electrodeposition voltage largely depends on the surface area ratio of the counter electrode and the working electrode. For example, using the same Pt counter electrode, the oxidation voltage stabilized at 0.78 V for cm^2-scale electrodes (Supplementary Fig. 1), while the voltages for mm^2-scale nerve cuff devices are lowered to 0.64 V.

After the completion of electrodeposition, each film was cycled 5 times between -0.8 to 0.1 V at 10 mV/s. As shown in Supplementary Fig. 2, it generates a very large reduction peak for the first cycle, indicating the incorporation of large amount of Na^+, while it seems that some of these cations were trapped in the polymer matrix because a relatively smaller peak was shown in the following oxidation scan. Additionally, similar current values were produced in all the subsequent scans and the peak positions turned to be stable after 4 cycles. The included photographs illustrate the status of the device at 0.1 V to show apparent effect of such an operation. As the scan started, the bilayer device was flat at 0.1 V without any movement. However, it started to show bending movement at the same voltage of 0.1 V after the completion of one cycle, and such movement was more and more significant as the proceeding of this process. The preconditioning ended up with a fully curled state, indicating that the device was ready to use for reliable actuation.

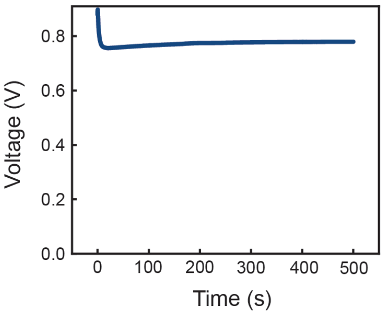

Supplementary Fig. 1 Electrochemical deposition of PPy(DBS).

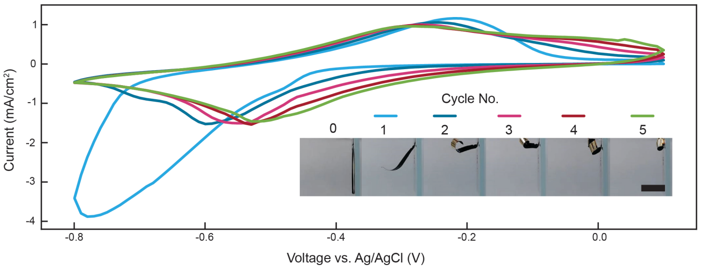

Supplementary Fig. 2 Cyclic voltammetry scans to precondition the PPy(DBS). The inserted photographs show the bending states at 0.1 V after each cycle. Scale bar: 5 mm.

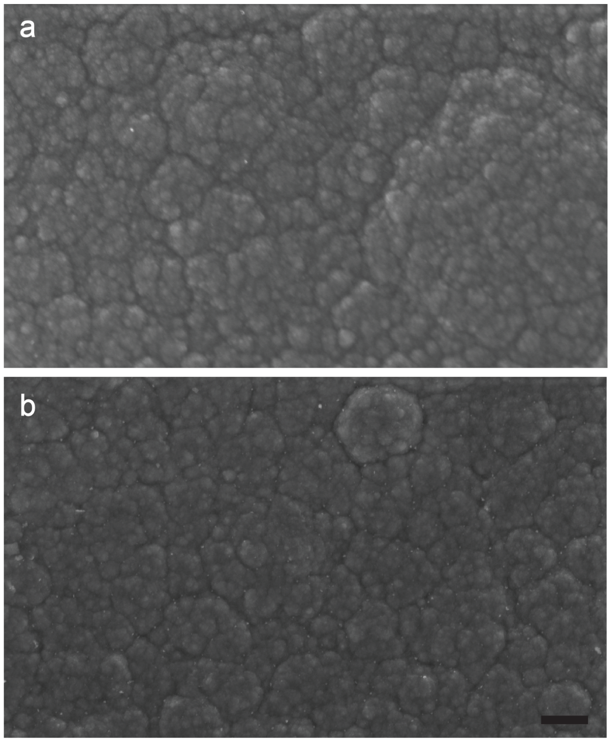

Supplementary Fig. 3 SEM of the PPy(DBS) before (a) and after (b) the preconditioning scan. Scale bar: 1 μm.

Supplementary section 2. Electrochemical impedance spectroscopy (EIS) characterization of PPy(DBS)

EIS is a powerful technique used to study intrinsic material properties and electrochemical responses. We therefore performed EIS to characterize the actuating material both before and after the conditioning treatment. Nyquist and Bode plots in Fig. 1f illustrate the impedance variations pre- and post-preconditioning. Our analysis employed a transmission line model, commonly employed to elucidate electrochemical processes in highly porous electrode materials. As depicted in Supplementary Fig. 4, these electrodes consist of two components: the base electrode and the porous electrode. Unlike planar electrodes, where reactions occur directly on the surface, reactions within the pores of porous materials are constrained. The limited access to the active interface for ions, attributed to the small pore size, results in ion diffusion becoming the dominant step.

The stepwise ion flux within a pore is described using a transmission line model, comprising parallel and serially connected elements[1], as depicted in Supplementary Fig. 4. R_0 characterizes the electrolyte resistance. $R_a||Q_1$ and $R_b||Q_2$ represent impedances on the outer surface of the pore (x=0) and on the base electrode at the end of the pore (x=L, where L is the pore depth), respectively. The transmission line along the pore is represented through repeating impedance elements, with R_1 signifying a local ohmic drop at each point of the transport channels, contingent

on electrolyte conductivity, and $\varsigma||Q_2$ describing the exchange of electrical charges at the interface due to Faradaic currents and polarization at the pore surface.

As demonstrated in Fig. 1f, the model aligns well with the measured spectra across all frequency regions, and Supplementary Table 1 summarizes the fit parameters. Following preconditioning, R_a and ς significantly decrease due to charge transfer reactions occurring on the active material. Q_1 and Q_2, representing the double-layer capacitance of the system, exhibit an increase. We reckon that the augmented charge transfer aligns with the results in Supplementary Fig. 2, where the film experiences increasing strain during preconditioning. It is likely that structural changes in the polymer during treatment impact the film's porosity and ion diffusivity[2].

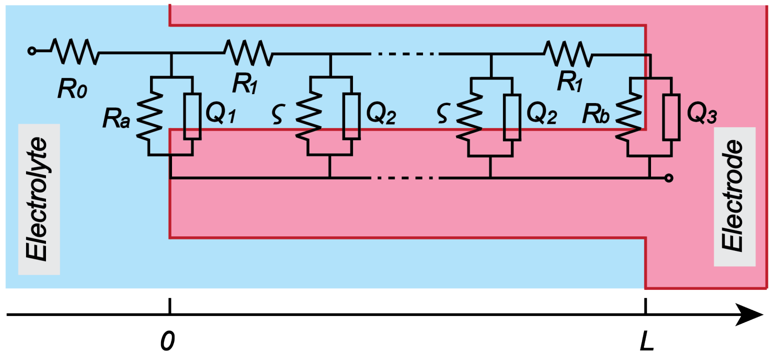

Supplementary Fig. 4 Scheme of the transmission line model used to fit the EIS results of PPy(DBS).

Supplementary Table 1. Fit parameters for the EIS of PPy(DBS) using the transmission line model.

**Samples**	**R_0 (Ω)**	**R_1 (Ω)**	**R_a (Ω)**	**Q_1 (S·μs$^\alpha$)**	**R_b (Ω)**	**Q_3 (S·μs$^\alpha$)**	**ζ (Ω)**	**Q_2 (S·μs$^\alpha$)**	**L**
**Before PreC**	79.4	358.3	242828	7.8 (α=1)	1000000	2.2 (α=0.9)	34504.7	0.02 (α=1)	0.06
**After Prec**	107.2	602.7	2046.9	145.0 (α=0.8)	1000000*	2.2* (α=0.9)	148.4	0.18 (α=1)	1

*: We find that these two parameters do not influence the agreement of the fitting, therefore we simply choose the same value as that used for the samples before PreC.

Supplementary Fig. 3. Electrochemical characterization of PPy(DBS)

Fig. 1g displays typical CV curves recorded at various scan rates in the voltage range of 0.1 to -1.1 V (vs. Ag/AgCl). The current modulation observed in the peak current regions is attributed to disturbance caused by the significant bending movement of the tested films. The curves for samples without any movement are shown in Supplementary Fig. 5, which feature smooth currents throughout the voltage window. The negative scan involves the incorporation of solvated Na^+ ions into the polymer matrix, while the positive current corresponds to the expulsion of these cations. Achieving the maximum actuating amplitude requires a completion of the electrochemical process during scanning, which becomes more challenging and necessitates higher voltages with

the increase of scan rates. This is evident from the gradually separated oxidation and reduction peaks at higher scan rates. The design of these actuators, particularly the thickness of the actuating material, largely influences the number of involved cations and, consequently, the final actuating performance.

Fig. 1h shows the calculated specific capacitance as a function of scan rates for PPy with varied thicknesses. In general, thicker samples exhibit higher capacitance values due to the involvement of more active sites. For example, at the scan rate of 25 mV/s, the specific capacitance values are 210, 445, 807 and 1198 mF/cm^2 for samples with thicknesses of 1.3, 6.7, 13.4 and 26.8 μm, respectively. All of the films show decrease in capacitance with increasing scan rates, particularly the 26.8-μm-thick ones, attributed to limited penetration of cations during a scan. We further conducted multistep amperometry tests by applying a series of progressively increased voltage steps, starting from -0.7 to 0.6V, with each step increment of 0.1 V and holding for 20 s. As shown in Fig. 1i, the positive current mainly occurs between -0.4 and 0 V, while the appearance of more negative current is observed between -0.3 and -0.6 V. The current generally returns to zero after a step change in voltage, but at specific voltages, it levels out to a non-zero value. This phenomenon can be attributed to the coexistence of both Faradaic and non-Faradaic processes[3].

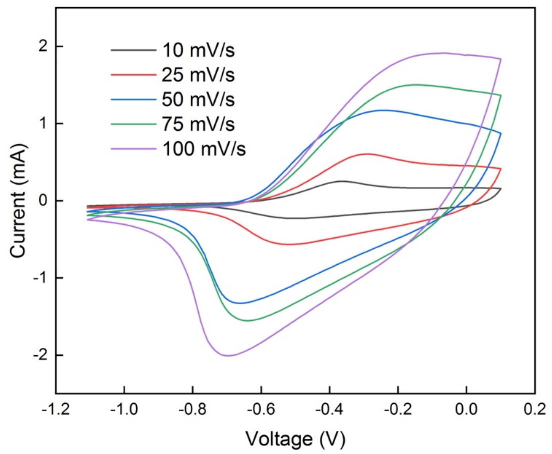

Supplementary Fig. 5 CV curves of PPy(DBS) in PBS. Samples were fixed on glass slides to prevent any movement during the test.

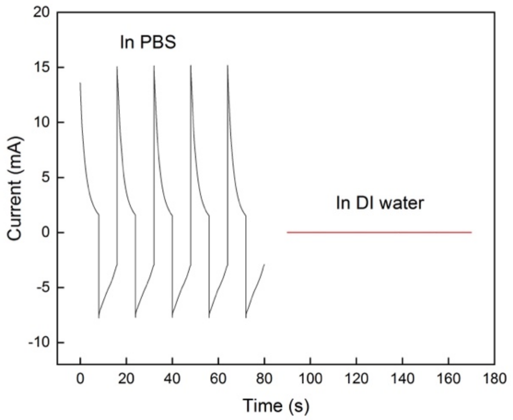

Supplementary Fig. 6 Performance comparison of devices tested in PBS and DI water.

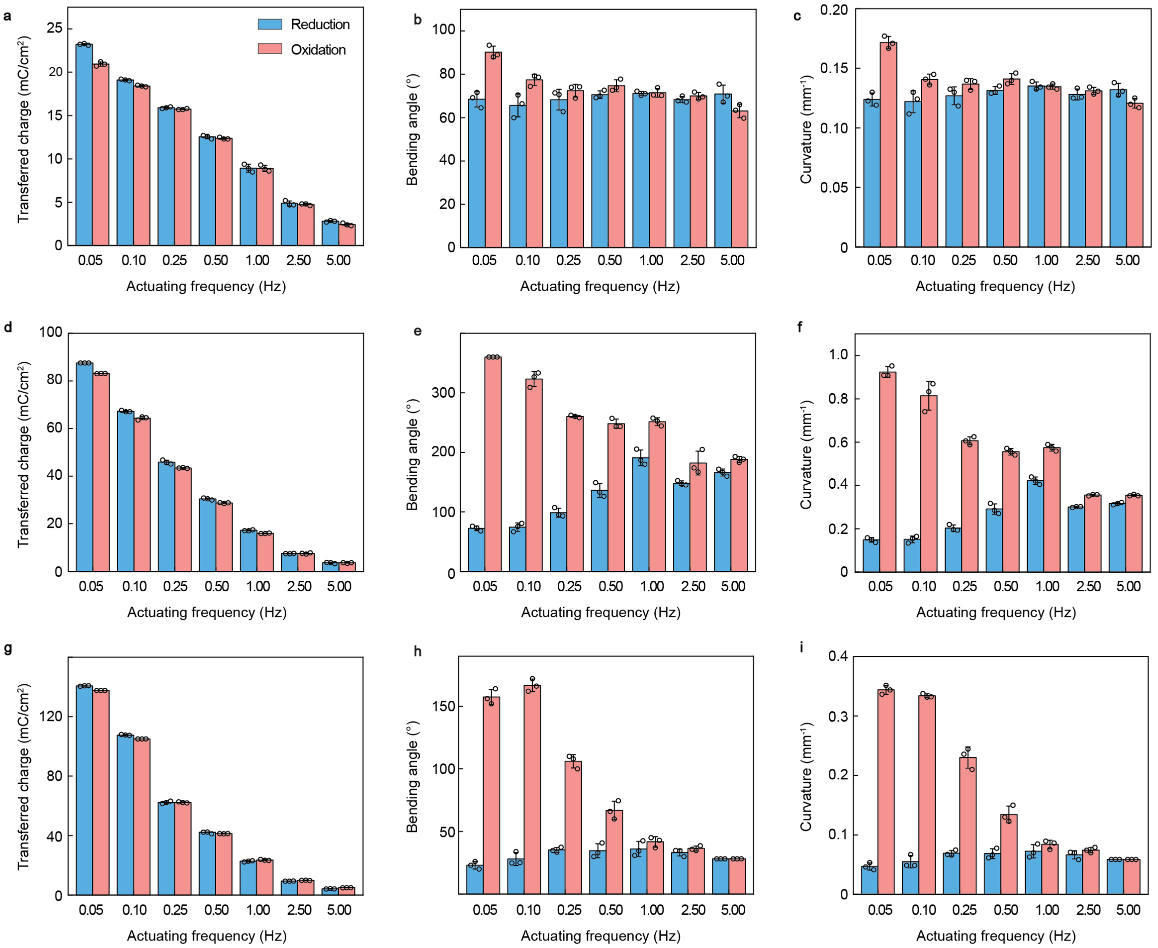

Supplementary Fig. 7 Evaluation of actuating performance of devices with varied PPy(DBS) thickness. a-c, 1.3 μm, d-f, 13.4 μm, g-i, 26.8 μm. The thicknesses of all other layers are the same. Data are shown as mean ± s.d. (*n*=3).

Supplementary section 4. Estimation of bending stiffness

We estimated the bending stiffness of our sample using the theory developed by Pister and Dong[4] for the elastic bending of laminated plates, which was derived based on the Föppl–von Kármán plate theory. For a plate consisting of bonded layers made of isotropic materials, its bending stiffness can be computed as

$$D = \frac{AC - B^2}{A}. (1)$$

The constants in Eq.(1) are

$$A = \sum_k \frac{E_k}{1 - \nu_k^2}(z_k - z_{k-1}),$$

$$B = \sum_k \frac{E_k}{1 - \nu_k^2} \frac{z_k^2 - z_{k-1}^2}{2},$$

$$C = \sum_k \frac{E_k}{1 - \nu_k^2} \frac{z_k^3 - z_{k-1}^3}{3},$$

where E_k and v_k are the Young's modulus and Poisson's ratio of the kth layer, respectively, and z_k is the distance from the top of the plate to the top of the kth plate lamina. Note that the expression of bending stiffness given in Eq.(1) has been simplified under the assumption that the Poisson's ratios are nearly constant across all layers[4].

In our case, we have:

Layer	E_k (GPa)	v_k	t_k (µm)
PaC	1.13	0.4	1.95
Ti	120	0.34	0.01
Au	79	0.415	0.1
PPy	0.15	0.35	6.7

Then, the bending stiffness according to Eq.(1) is D=21 Pa·mm^3, which is equivalent to a single PPy plate with a thickness of 11.4 µm or a PDMS with a thickness of 40 µm.

Supplementary section 5. The effects of PaC and PPy(DBS) thickness on actuating performance

During our demonstration in Fig. 3g and Supplementary Video 4, the top section of the device was firmly clamped to ensure stable recordings, thus maintaining a consistent distance between the nerve phantom and the device and causing a relatively loose interaction for the first circle on the left. However, in a surgical setting, adjusting the contact is easily achievable by gently lifting the device slightly. Additionally, it was noted that the small tip area may overlap with itself. If such overlap occurred during implantation surgery, a gentle manoeuvre using a blunt tool would be employed to carefully nudge the tip forward, thereby separating it from the overlapped segment. Our ultimate aim is to achieve a large bending amplitude for an intimate nerve interface at a very low voltage, or ideally even when the power supply is turned off, to minimise noise during electrophysiology recording. To achieve this goal, we first maximised the actuator area and optimised the thickness of PaC and PPy(DBS). We first evaluated devices with a fixed PPy(DBS) thickness of 4 µm, while varying the PaC thickness from 2 to 4 µm. A series of voltage steps were applied and maintained for 10 seconds at each step. The 4 µm-thick PaC samples exhibit insufficient bending at applied voltages (Supplementary Fig. 8a). In contrast, the 3 µm-thick PaC devices display noticeable bending behaviour at 0.2 V and show increased bending with higher voltages (Fig. 3h and Supplementary Fig. 8b). Indeed, this performance could be further enhanced with a slightly thicker PPy(DBS) (Fig. 3h and Supplementary Fig. 8c). Notably, further reducing the PaC thickness to 2 µm results in significant enhancement in bending amplitude,

achieving a radius of 0.76 mm at 0 V, and a higher voltage allows the radius to reach 0.4 mm (Fig. 3h and Supplementary Fig. 8d).

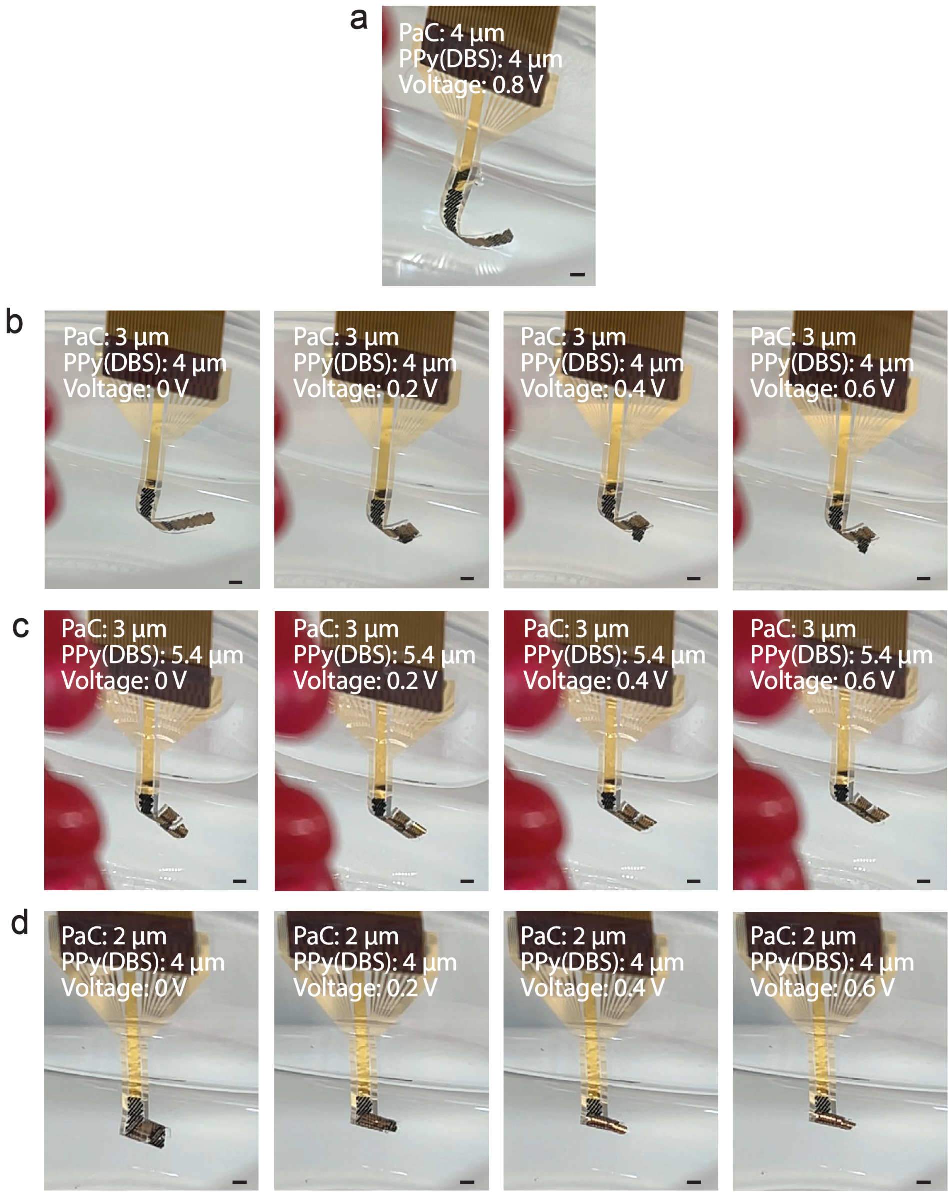

Supplementary Fig. 8 Photographs showing actuating performance of various devices. The detailed information about parameters of the devices including thickness of PaC and PPy(DBS) and the applied voltages are listed in each photograph. Scale bar: 2 mm.

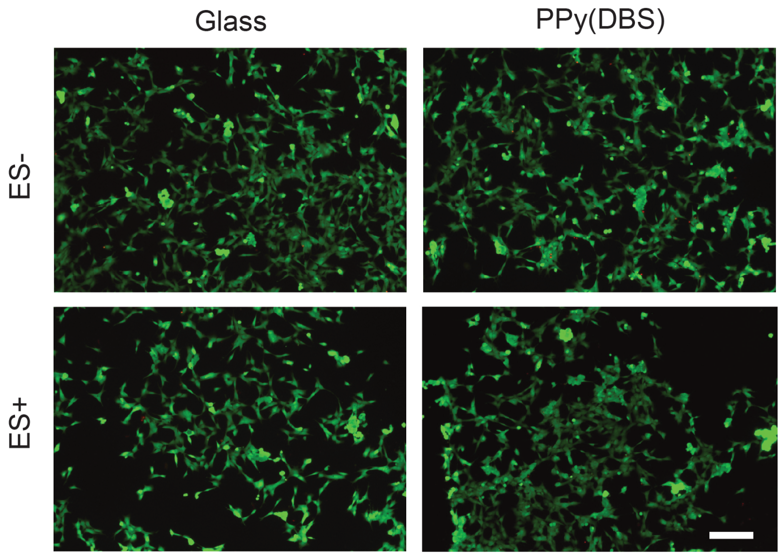

Supplementary Fig. 9 Live/Dead staining of PPy and the control groups of glass slides. Green: live cells. Red: dead cells. ES- and ES+ mean without and with the application of voltage stimulation. Scale bar: 200 μm.

References

1. Bisquert, J. *et al.* Doubling exponent models for the analysis of porous film electrodes by impedance. Relaxation of TiO_2 nanoporous in aqueous solution. *J Phys Chem B* **104**, 2287–2298 (2000).
2. Marchesi, L., Simões, F. R., Pocrifka, L. A. & Pereira, E. C. Investigation of polypyrrole degradation using electrochemical impedance spectroscopy. *J Phys Chem B* **115**, 9570–9575 (2011).
3. Biesheuvel, P. M., Porada, S. & Dykstra, J. E. The difference between Faradaic and non-Faradaic electrode processes. *arXiv preprint arXiv:1809.02930* (2018).
4. Pister, K. S. & Dong, S. B. Elastic bending of layered plates. *Journal of the Engineering Mechanics Division* **85**, 1–10 (1959).
